# Targeting the Immune System for Pulmonary Inflammation and Cardiovascular Complications in COVID-19 Patients

**DOI:** 10.3389/fimmu.2020.01439

**Published:** 2020-06-23

**Authors:** Serena Colafrancesco, Rossana Scrivo, Cristiana Barbati, Fabrizio Conti, Roberta Priori

**Affiliations:** Rheumatology Unit, Department of Clinical, Internal, Anesthesiological and Cardiovascular Sciences, Sapienza University of Rome, Rome, Italy

**Keywords:** COVID-19, interleukin-6, interleukin-1, JAK inhibitors, hydroxychloroquine, ARDS, coagulation

## Abstract

In December 2019, following a cluster of pneumonia cases in China caused by a novel coronavirus (CoV), named severe acute respiratory syndrome coronavirus 2 (SARS-CoV-2), the infection disseminated worldwide and, on March 11th, 2020, the World Health Organization officially declared the pandemic of the relevant disease named coronavirus disease 2019 (COVID-19). In Europe, Italy was the first country facing a true health policy emergency, and, as at 6.00 p.m. on May 2nd, 2020, there have been more than 209,300 confirmed cases of COVID-19. Due to the increasing number of patients experiencing a severe outcome, global scientific efforts are ongoing to find the most appropriate treatment. The usefulness of specific anti-rheumatic drugs came out as a promising treatment option together with antiviral drugs, anticoagulants, and symptomatic and respiratory support. For this reason, we feel a duty to share our experience and our knowledge on the use of these drugs in the immune-rheumatologic field, providing in this review the rationale for their use in the COVID-19 pandemic.

## Introduction

In December 2019, an outbreak of an unknown infectious disease denominated coronavirus (CoV) disease 2019 (COVID-19), caused by a novel CoV named severe acute respiratory syndrome coronavirus 2 (SARS-CoV-2) (1), was reported in Wuhan, the capital of Hubei Province, People's Republic of China (2). Subsequently, the infection rapidly expanded worldwide, and the World Health Organization consequently declared it a pandemic on March 11th, 2020.

Despite a favorable clinical course in most patients, a significant amount of severe interstitial pneumonia cases related to COVID-19 have been described up until now. The rate of mortality is around 2%, and acute respiratory distress syndrome (ARDS) is the major complication (3). As observed in previous epidemics caused by other types of CoV, such as severe acute respiratory syndrome-CoV (SARS-CoV) and the Middle East respiratory syndrome-CoV (MERS-CoV), highly pathogenic CoV poses a substantial threat to public health. During the 2002–2003 epidemic, SARS-CoV infected ~8,400 individuals and had a 9.6% overall mortality rate (WHO Cumulative number of reported probable cases of SARS in 2003); in 2012, MERS-CoV infected 1,936 individuals and had a mortality rate of around 36% [WHO: Middle East respiratory syndrome coronavirus (MERS-CoV). http://www.who.int/emergencies/mers-cov/en/]. Following the eruption of COVID-19 in China, the infection disseminated worldwide, and in February 2020 the first European cases of COVID-19 were described in Northern Italy (4).

Similar to previous CoV outbreaks, the clinical spectrum of COVID-19 ranges from asymptomatic infection to severe respiratory failure, especially described in the elderly and patients with comorbidities (5).

What is currently known in the ongoing pandemic is that humans affected by COVID-19 with fatal outcome experience a deregulated immune response that results in exuberant inflammation and lethal disease (2). Similarly, in most severe cases of SARS-CoV and MERS-CoV, high serum levels of several pro-inflammatory cytokines were found (6, 7). However, differently from previous CoV infections, SARS-CoV-2 is extremely contagious (8), and the progression to ARDS is dramatic and quick in some cases. The specific host factors driving this severe lung pathology are relatively unknown, but, in a retrospective analysis of adult inpatients with COVID-19 from two hospitals in Wuhan, older age, elevated D-dimer levels (>1 μg/L), and high sequential organ failure assessment score on admission emerged as potential risk factors for poor prognosis (9).

Some biological mechanisms are deemed to be pathogenic, including a rapid virus replication, a predominant CoV infection of the airway and/or alveolar epithelial cells, a delayed interferon (IFN) response, and the accumulation of monocytes, macrophages, and neutrophils in the alveoli (10). During ARDS, massive damage in lung microvascular endothelial and epithelial cells occurs with the resulting accumulation of protein-rich edema in the alveoli and infiltration of neutrophils, macrophages, and red blood cells (11). In particular, local production of pro-inflammatory molecules mediated by adaptive and innate immune cells, together with activated epithelial cells, contribute to exaggerated recruitment of inflammatory cells and support the local release of proteases and oxidants responsible for disruption of the blood-alveolar barrier, pulmonary edema, intrapulmonary hemorrhage, and severely impaired gas exchange (10). The inflammation driving ARDS, if not locally controlled, may lead to a severe systemic inflammatory response syndrome (SIRS), possibly resulting in multi-organ failure (12). This massive uncontrolled inflammatory response is likely the main cause of the dramatic deterioration observed in some COVID-19 patients. In these cases, the use of immunosuppressive/immuno-modulating drugs seems to offer a better clinical outcome, as do antiviral drugs, symptomatic and respiratory support, and anticoagulants. Indeed, it has recently emerged that a hallmark of severe COVID-19 is also the occurrence of coagulopathy, with 71.4% of patients who die meeting the criteria for disseminated intravascular coagulation (DIC) (13). This is characterized by a pro-thrombotic state with evidence of elevated D-dimer and fibrinogen levels, low anti-thrombin levels, and pulmonary congestion with microvascular thrombosis (13). Acro-ischemia is a frequent presentation of this complication being associated with a significant rate of death (14). Activation of coagulation pathways can drive overproduction of proinflammatory cytokines, which leads to multiorgan injury (15). For instance, although thrombin is mainly charged with the promotion of clot formation, it also exerts multiple cellular effects and augments inflammation via proteinase-activated receptors (PARs) (16). However, the increased vascular coagulation occurring in COVID-19 patients is more similar to a lung-centric intravascular coagulopathy (PIC) than it is to the classical DIC (17). This peculiar presentation seems related to a macrophage activation syndrome (MAS)-like intrapulmonary inflammation, which differs from the classical MAS observed along the course of inflammatory or infectious conditions (17). Thus, inflammation can activate the coagulation cascade and down-regulate anticoagulant mechanisms (18). Increased circulating D-dimer concentrations reflect the ongoing pulmonary vascular bed thrombosis and, together with elevated cardiac enzyme concentrations, secondary to ventricular stress induced by pulmonary hypertension is an early feature of PIC related to COVID-19 (19). The presence of increased D-dimer levels raises concerns regarding the coexistence of venous thromboembolism, which further deteriorates pulmonary function. Thus, the administration of a prophylactic dose of low molecular weight heparin (LMWH) is currently recommended and seems to be beneficial not only to prevent vascular complications but also to reduce the inflammatory reaction due to its additional anti-inflammatory properties (20).

Of interest, three Chinese COVID-19 patients presenting thrombotic events tested positive for anti-phospholipid antibodies such as anti-cardiolipin IgA antibodies and anti–β2 glycoprotein I IgA and IgG antibodies (21). In patients with autoimmune or autoinflammatory conditions, such as catastrophic anti-phospholipid syndrome (cAPS) and adult-onset Still disease (AOSD), dramatic situations such as SIRS or DIC are occasionally observed. The similarities shared by these conditions, known to be gathered under the term of “hyperferritinemic syndromes” (22), led us to suggest the inclusion of COVID-19 in this spectrum of conditions (23). The presence of uncontrolled inflammation supports the rationale to adopt immune suppressive treatments targeting specific pro-inflammatory molecules.

Interestingly, preliminary data from Lombardy, the region in Northern Italy with the highest incidence of COVID-19 cases, do not show an increased risk of respiratory or life-threatening complications from SARS-CoV-2 in immunosuppressed patients with chronic arthritis compared with the general population, and a similar experience were reported with SARS-CoV and MERS-CoV (24).

**Figure 1:**
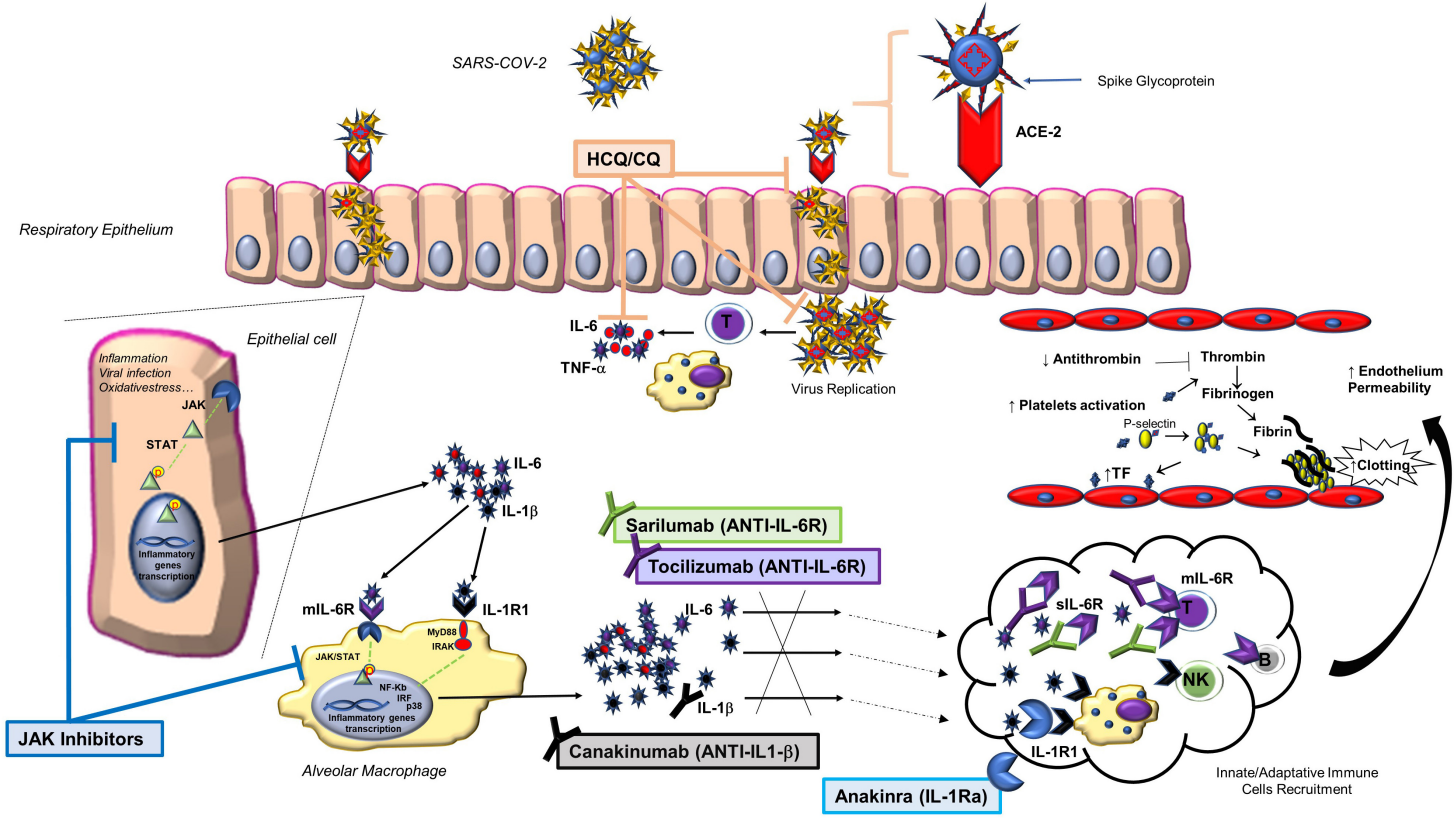
Main biological pathways activated by SARS-CoV-2 infection and treatment strategies to block them through anti-rheumatic drugs. Following SARS-CoV-2 binding to the ACE-2 host receptor via the spike glycoprotein, a series of events occur within the affected cell, including the activation of the JAK/STAT pathway and the release of pro-inflammatory cytokines, such as IL-1β and IL-6. This finding, together with the observation that IL-6 is largely involved in the lung damage that may complicate the infection, led to include the use of some anti-rheumatic drugs (anti-IL6: tocilizumab and sarilumab; antimalarials: chloroquine and hydroxychloroquine) in various treatment protocols. Other anti-rheumatic drugs may be of interest in the treatment of COVID-19 (IL-1 and JAK inhibitors). ACE-2, angiotensin-converting enzyme 2; CQ, chloroquine; HCQ, hydroxychloroquine; IL-1Ra, IL-1 receptor antagonist; IL-1R1, type 1 IL-1 receptor; JAK, Janus kinase inhibitor; mIL-6R, transmembrane IL-6 receptor; NK, natural killer cell; p, phosphate; SARS-CoV-2, severe acute respiratory syndrome coronavirus 2; sIL-6R, soluble IL-6 receptor; STAT, signal transducers and activators of transcription; TF, tissue factor; TNF, tumor necrosis factor.

Being very familiar with the use of anti-rheumatic immunosuppressive therapies, including those recently proposed for the treatment of COVID-19, we deemed it proper to share our experience and knowledge on these drugs, i.e., the inhibitors of interleukin 6 (IL-6) and the antimalarials chloroquine (CQ) and hydroxychloroquine (HCQ). In this review, we provide the rationale for their possible usefulness in the COVID-19 pandemic; furthermore, we discuss other rheumatological treatments of potential interest, including IL-1 and Janus kinase (JAK) inhibitors (Figure 1).

### IL-6 Inhibitors

#### The Role in Rheumatic Diseases

Different cell types, mainly T lymphocytes and macrophages, produce IL-6, a pleiotropic cytokine involved in the regulation of immune response, hematopoiesis, and inflammation through its binding to transmembrane IL-6 receptor α (mIL-6R) as well as soluble IL-6R (sIL-6R) (25). IL-6 induces the proliferation and differentiation of T and B cells, but it is also directly involved in systemic and local inflammation targeting hepatocytes, hematopoietic progenitor cells, and fibroblasts. Systemically, IL-6 induces fever, fatigue, and anorexia as well as an increase in C-reactive protein (CRP) (26). In patients with rheumatoid arthritis (RA), IL-6 promotes angiogenesis in the affected joints and induces the differentiation of osteoclast precursor cells to mature osteoclasts, which results in the bone absorption and joint destruction typical of the disease (27). Because of this pathological role, IL-6 represented an attractive therapeutic target in RA, and tocilizumab, a humanized anti-IL-6R monoclonal antibody of the IgG1 class, was approved in Europe in 2009 as intravenous injections (and in 2013 as subcutaneous injections) for the treatment of adult patients with RA. Subsequently, tocilizumab gained approval for the treatment of juvenile idiopathic polyarthritis, including the systemic form (SJIA) and, more recently, for the treatment of giant cell arteritis (GCA) and severe or life-threatening cytokine release syndrome (CRS), which may develop as a side effect of chimeric antigen receptors (CAR) T-cell therapies or because of infectious stimuli (EMA website; European public assessment reports).

As regards to RA, tocilizumab was tested in several randomized, controlled trials (RCTs) of ≥24 weeks' duration involving around 7,000 RA patients (28). These RCTs demonstrated the sustained efficacy of the drug, as monotherapy or combination therapy, in terms of clinical and radiographic outcomes, as well as health-related quality of life in both early-stage and established RA. Common adverse reactions were nasopharyngitis, upper respiratory tract infections, injection site reactions, increased liver enzymes, hypercholesterolemia, headache, neutropenia, and increased LDL cholesterol; uncommon adverse reactions were diverticular perforations as complications of diverticulitis. The recommended intravenous dosage of tocilizumab is 8 mg/kg once every 4 weeks (doses >800 mg not recommended), while the recommended subcutaneous dosage is 162 mg once weekly (https://www.ema.europa.eu/en/documents/product-information/roactemra-epar-product-information_en.pdf). As of June 2017, EMA released marketing authorization for another biological agent targeting IL-6R, sarilumab, a fully human IgG1 monoclonal antibody, for the treatment of RA in adults (EMA website; European public assessment reports). When the binding kinetics and functional activity of tocilizumab and sarilumab were compared, the latter bound to mIL-6R and sIL-6R with higher affinity than tocilizumab and inhibited IL-6R activation and IL-6-induced cell proliferation at lower concentrations than tocilizumab (29). The recommended dose of sarilumab is 200 mg once every 2 weeks, administered as a subcutaneous injection. A reduced dose of 150 mg once every 2 weeks is recommended for the management of treatment-emergent neutropenia, thrombocytopenia, and elevated liver enzymes (EMA; Kevzara: summary of product characteristics 2017; http://www.ema.europa.eu/). In the 2019 update of the European League Against Rheumatism (EULAR) recommendations for the management of RA, the use of a biological agent (including tocilizumab and sarilumab) is encouraged as second-line therapy in the presence of poor prognostic factors, and IL-6 inhibitors should be preferred over the other biological agents in patients who cannot use concomitant conventional treatment (30).

#### The Role in Pulmonary Inflammation

It is of note that 10% of RA cases display an interstitial lung disease (ILD) (31), which is also reported in 10–40% of patients with connective tissue diseases (32). Therefore, some data are available on the possible benefit of IL-6 inhibition in lung inflammatory conditions. In patients with RA and ILD, tocilizumab demonstrated an acceptable safety profile and efficacy on the stabilization of lung involvement (33). A favorable outcome in ILD following the use of tocilizumab was also observed in patients with undifferentiated autoinflammatory syndromes (34, 35) and systemic sclerosis. In this last condition, tocilizumab demonstrated efficacy on lung involvement in up to 46% of patients, especially those with early disease onset (36).

In the context of lung inflammation, IL-6 production is mainly mediated by innate and adaptive immune cells, along with local “activated” epithelial cells, which participate in the inflammatory response (37). In lung tissue, IL-6 production is induced by different stimuli including allergens, viral infections, and “injurious” mechanical ventilation (38). However, the role of IL-6 in lung injury is still unclear, and both protective and detrimental effects have been described (39).

During H1N1 influenza infection, IL-6 exhibits a pleiotropic role, equally regulating the innate and adaptive immune response (40, 41), and its increased serum level has been proposed as a hallmark of pneumonia severity in more seriously ill H1N1 patients (42). Furthermore, in patients with ARDS, the hyper-expression of IL-6 at plasma and bronchoalveolar lavage (BAL) fluid level seems associated with a poor disease outcome (43). In line with what observed in humans, in murine models of ARDS, the deficiency of IL-6 is associated with a decrease in BAL cellular inflammation and less severe lung damage (39). In addition, *in vitro* data demonstrate that IL-6 is responsible for a significant increase in endothelial permeability with consequent recruitment of inflammatory cells at the alveolar level (39). Additionally, several reports indicate that IL-6 primarily contributes to increments in respiratory system resistance, and its pathogenic role in several respiratory disorders, such as asthma and chronic obstructive pulmonary disease (COPD), has been described (44). High IL-6 blood concentrations are also associated with vascular remodeling and pulmonary hypertension, hyperplasia and hypertrophia of the vascular muscular wall, and decreased endothelium-dependent vascular wall relaxation (45). It is of note that human airway smooth muscle cells are capable to produce IL-6, too (46), and this production is likely able to cause airway remodeling in asthmatic subjects (47).

Despite its possible pathogenic role in lung inflammatory diseases, IL-6 is also a crucial regulator of the balance among fibroblasts, macrophages, and epithelial lung cells (48). Specifically, since IL-6 seems able to participate in the resolution of inflammation by the suppression of TGF-β production, a prolonged therapeutic blockade of this cytokine pathway in lung inflammatory conditions needs to be carefully considered. A further reason requiring attention, particularly in the choice of the timing of IL-6 inhibition, derives from the observation that throughout infections IL-6 might reduce inflammation preventing virus-induced lung epithelial cells apoptosis and promoting macrophage recruitment within the lung and virus-infected cells phagocytosis (48).

#### The Role in Cardiovascular Risk and Coagulation

Inflammation and thrombosis share common signaling pathways, and the inflammatory response promotes the activation of the clotting cascade and platelets. Inflammation plays a major role in cardiovascular complications where IL-6, together with other cytokines, establishes a prothrombotic state by disabling the natural inhibitors of hemostasis and natural anticoagulants in addition to other external factors (49).

In chronic inflammatory rheumatic diseases, increased cardiovascular risk, mainly related to accelerated atherosclerosis, has been documented (50, 51). In this context, IL-6 participates in the formation of atherosclerotic plaques (52), and, accordingly, individuals with a variant in the IL-6R were found to have a decreased risk for coronary heart disease (53). Thus, in patients with RA IL-6 blocking is a reasonable approach both to decrease disease activity and to reduce cardiovascular risk. Yet, the use of tocilizumab in patients with RA is associated with an increased circulating concentration of LDL and altered expression of LDL hepatic receptor, which could adversely affect cardiovascular risk (54). However, this does not seem significantly higher compared to the other biological agents (55), and, indeed, not only IL-6 inhibition seems associated with a cardiovascular protective effect, but it is currently a therapeutic option in GCA and Takayasu arteritis (56). Furthermore, in GCA IL-6 is considered a sensitive biomarker of disease activity (57, 58).

Aside from a pro-inflammatory role toward vascular endothelial cells, IL-6 may favor hypercoagulation albeit at a lesser extent than IL-1 and IL-8 (59). Importantly, IL-6 is one of the highest circulating cytokines expressed in patients with sepsis-induced DIC (60), and it is considered an early predictor of DIC in patients with sepsis (61). Proof of IL-6 interferences with the coagulation cascade also comes from studies testing the inhibition of this molecule in inflammatory conditions. In patients with RA treated with tocilizumab, a decrease of factor XIII, which is involved in thrombotic complications, has been observed (62). Accordingly, in mice models of cancer-related cachexia characterized by a hypercoagulable state, silencing of IL-6 significantly attenuated the increased thrombin generation, with similar trends for fibrinogen and tissue factor pathway inhibitor (63). Finally, endothelium activation is another major mechanism in thrombotic events that may be affected by IL-6, one of the cytokines able to change the normal anticoagulant and profibrinolytic properties of endothelial cells; this consequently induces an activated state that fosters thrombus formation and stalls fibrinolysis. Of note, an increased activation of endothelial markers has been associated with the presence of ARDS (64).

Thus, it is likely that, in severe cases of COVID-19, the development of DIC derives from multiple factors orchestrated by pro-inflammatory molecules, including IL-6, that concur in damaging blood vessels, interfering with coagulation, and inducing endothelial cell activation. In line with this evidence, IL-6 inhibition may be beneficial also for cardiovascular thrombotic complications occurring in patients with COVID-19.

#### The Role in COVID-19 Patients

In China, tocilizumab was administered to 21 patients diagnosed as severe or critical COVID-19 in addition to what was considered standard therapy [Diagnosis and treatment protocol for novel coronavirus pneumonia (7th interim edition), China NHCOTPSRO]. Severity in adults was defined if any of the following conditions were met: a) respiratory rate ≥30 breaths/min, b) SpO2 ≤ 93% while breathing room air, c) PaO2/FiO2 ≤ 300 mmHg, and d) relevant progression (>50%) at chest radiograph in 24–48 h. A critical case was diagnosed if a respiratory failure requiring mechanical ventilation or shock or organ failure occurred. In most patients, tocilizumab demonstrated a dramatic efficacy with complete resolution or significant amelioration of fever, CT scans imaging, leukopenia, and reduction in the levels of CRP. In 75% of cases, oxygen intake was lowered, and in one case it was discontinued. Nineteen patients (90.5%) were discharged on average 13.5 days after the treatment (65). In another study, tocilizumab was administered in eight of 15 hospitalized patients with COVID-19 in combination with methylprednisolone. Although tocilizumab dramatically reduced CRP levels, of the four critically ill patients who received one single dose of the drug, three died, and the remaining one worsened. Compared to the other patients a persistent and dramatic increase of IL-6 was observed in these four patients who failed treatment, suggesting that for critically ill patients with elevated circulating IL-6 levels, the repeated dose of tocilizumab should be considered (66). In addition, some single case reports supporting the use of IL-6 inhibitors in severe COVID-19 have been published (67–69).

In Italy, following the dramatic spread of SARS-CoV-2, we are experiencing a true emergency in our hospitals, especially in the intensive care units, hosting patients with SIRS and ARDS. SIRS is determined by a true cytokine storm mainly amplified by IL-6. As tocilizumab is approved for CRS associated with CAR-T therapy, the rationale supporting its use in COVID-19 systemic complications is even stronger.

Data from recent retrospective Italian cohorts provided contradictory results (70, 71). A study evaluating the efficacy of tocilizumab (400 mg iv 24 h apart in case of respiratory worsening) in patients with severe COVID-19 pneumonia and hyperinflammatory features (median CRP 156 mg/L (IQR 100–208) did not show a dramatic improvement compared to the standard of care (*n* = 32 and *n* = 33, respectively). Specifically, after 28-days of follow-up, a similar improvement in clinical conditions in both tocilizumab and standard of care groups was reported (69 vs. 61%, respectively). Despite the presence in tocilizumab group of a lower mortality rate (15%), the difference was not statistically significant (70). In a different study, including 33 patients treated with tocilizumab 400 mg intravenously and 27 treated with tocilizumab 324 mg subcutaneously, a greater survival rate and a significantly lower rate of death was observed with respect to the standard of care (*n* = 23) (71). However, all the previous studies are limited by their retrospective nature and a comparison between them cannot be easily performed due to differences at baseline in clinical features and variances in concomitant therapies.

Hopefully, data from clinical trials will provide stronger evidence. One of the first clinical trials on the use of tocilizumab in patients with COVID-19 was started in Italy (TOCIVID-19, NCT04317092). The study was promoted by the National Cancer Institute of Naples and involved the National Institute for the Infectious Diseases “L. Spallanzani,” IRCCS (Rome). The “L. Spallanzani” group also released recommendations for COVID-19 clinical management, being the first Italian hospital to admit and manage patients affected by COVID-19. However, due to very limited clinical evidence, they should be considered as expert opinions, which may be subject to change depending on newly produced data. According to these recommendations, patients affected by respiratory symptoms, clinically unstable, not in critical conditions, as well as critical patients should be treated with tocilizumab 8 mg/kg (maximum 800 mg/dose) single-dose intravenously (1-h infusion); in the absence or with poor clinical improvement, a second dose should be administered after 8–12 h. According to these recommendations, tocilizumab administration should be guided by the presence of one or more of the following selection criteria: a) PaO2/FiO2 ratio <300 mmHg, b) rapid worsening of respiratory gas exchange with or without the availability of non-invasive or invasive ventilation, and c) IL-6 levels >40 pg/mL (if not available, D-dimer levels >1,000 ng/mL). Concomitant supportive and anti-viral therapy should be administered (72).

To date, 45 different clinical trials on the use of tocilizumab in patients with COVID-19 are ongoing worldwide (ClinicalTrials.gov; EU Clinical Trial Registry; Chinese Clinical trial registry; Iranian Registry of Clinical trials, Japan Pharmaceutical Information Center Clinical Trials). The Swiss drugmaker Roche has launched one of the largest studies in different countries in Europe and the USA (NCT04320615).

Similarly, 14 clinical trials evaluating the efficacy and safety of intravenous or subcutaneous administration of sarilumab are ongoing in different countries (ClinicalTrials.gov; EU Clinical Trials Register). Among them, one of the largest studies, promoted by Sanofi-Aventis, is recruiting patients from Europe, Canada, Japan, and Russia (EU Clinical Trials Register: 2020-001162-12).

In Italy, a specific protocol was released by the Italian Society of Infectious and Tropical Diseases, Lombardy Region Section, where the epidemic started (https://www.simit.org/images/documenti/Linee%20guida%20SIMIT%20LAZIO%20SARS%20CoV%202%20maggio%202020.pdf). Lombardy has been the area of Italy most affected by COVID-19 and the first in which hospital organization was reconfigured both in terms of spaces and medical staff (73). Facing an exponential growth of hospitalized individuals with COVID-19 led to share experience to optimize the outcome of the disease management. Table 1 shows the comparison between this protocol and the one provided by the “Spallanzani” Institute, both stratified according to the severity of the clinical conditions of the patients. Only RCTs will provide indications on the better drug regimen in this clinical setting.

**Table 1:** Italian Recommendations for Covid-19 treatment: comparison between Spallanzani recommendations and the Italian Society of Infectious and Tropical Diseases Lombardy Region Section (North of Italy) protocol.

**National institute for the infectious diseases “L. Spallanzani”,** **IRCCS recommendations for COVID-19 clinical management**			**Therapeutic protocol for COVID-19 by the italian society of infectious and tropical diseases lombardy region section (north of Italy)**		
**Illness severity**	**Antiviral/immunotherapy**	**Supportive therapy**	**Illness severity**	**Antiviral/immunotherapy**	**Supportive therapy**
Asymptomatic or mild infection	None	Symptoms control	Asymptomatic	None	(Clinical monitoring)
Stable patient presenting with respiratory and/or systemic symptoms (MEWS <3)	Lopinavir/ritonavir^*^ 200/50 mg tablets, 2 tablets q12h, during 14 days **and** HCQ phosphate^**^ 400 mg tablets, 1 tablet q12 as loading dose, followed by 200 mg tablets, 1 tablet q12, during 10 days, **or** CQ phosphate^**^ 250 mg tablets, 2 tablet q12, during 10 days	Symptomatic Oral rehydration Consider broad-spectrum antimicrobial therapy Prompt availability of O2, in case of necessity	Mild respiratory symptoms in patients <70 years old and/or no risk factors (diabetes, heart disease, chronic obstructive pulmonary disease), negative chest radiograph	None	Symptomatic treatment
Patient affected by respiratory symptoms, clinically unstable, not in critical conditions (MEWS 3-4)	**Remdesivir**^**°**^ (GS-57324), once daily intravenously: 200 mg loading dose, followed by 100 mg daily maintenance dose, during 10 days, **or (if Remdesivir not available)** Lopinavir/ritonavir^*^ 200/50 mg tablets, 2 tablets q12h, during 28 days **and** HCQ phosphate^**^ 400 mg tablets, 1 tablet q12 as loading dose, followed by 200 mg tablets, 1 tablet q12, during 10 days, **or** CQ phosphate^**^ 250 mg tablets, 2 tablet q12, during 10 days **and** **Tocilizumab**^∧^ 8 mg/kg (maximum 800 mg/dose), single-dose intravenously (1-h infusion); in the absence or with poor clinical improvement a second dose should be administered after 8–12 h	O2 administration Antimicrobial therapy Oral or intravenous rehydration Consider systemic steroids administration in case of clinical signs suggesting an incipient worsening of respiratory functions (steroids mandatory if tocilizumab is used): (methylprednisolone 1 mg/Kg daily intravenously for 5 days, followed by 40 mg daily for 3 days and, lastly, 10 mg daily for 2 days, or dexamethasone 20 mg daily intravenously for 5 days, followed by 10 mg daily for 3 days and lastly 5 mg daily for 2 days)	Mild respiratory symptoms in patients more than 70 years old and/or presence of comorbidities or increased risk of mortality Moderate respiratory symptoms and/or evidence of pneumonia at chest radiograph	**Lopinavir/ritonavir** 200/50 mg 2 tablets BID + **CQ phosphate** 500 mg BID for 20 days **or** **HCQ phosphate** 200 mg BID Alternative regimen: **Darunavir** 800 mg 1 tablet QD + **ritonavir** 100 mg 1 tablet QD **or** **darunavir/cobicistat** 800/150 mg QD (for 5 to 20 days, according to clinical evolution) If BCRSS score ≥2: **Dexamethasone** 20 mg per day for 5 days, then 10 mg per day for 5 days **and/or** **Tocilizumab:** 8 mg/kg, maximum 3 infusions (maximum dosage per infusion 800 mg); second infusion after 8–12 h; in case of inadequate response eventually third infusion after 16–24 h	Symptomatic treatment: O2 administration
Critical patient (MEWS>4)	**Remdesivir**^**°**^ (GS-57324), once daily intravenously: 200 mg loading dose, followed by 100 mg daily maintenance dose, during 10 days, **or (if Remdesivir not available)** Lopinavir/ritonavir^*^ 200/50 mg tablets, 2 tablets q12h, during 14 days **and** HCQ phosphate^**^ 400 mg tablets, 1 tablet q12 as loading dose, followed by 200 mg tablets, 1 tablet q12, during 10 days, **or** CQ phosphate^**^ 250 mg tablets, 2 tablet q12, during 10 days **and** **Tocilizumab**^∧^ 8 mg/kg (maximum 800 mg/dose), single-dose intravenously (1-h infusion); in the absence or with poor clinical improvement a second dose should be administered after 8–12 h	Gold standard: early protective mechanical ventilation Antimicrobial therapy Intensive care and monitoring as indicated by hospital protocols Systemic steroid therapy in case of ARDS/severe respiratory failure (steroids mandatory if tocilizumab is used): methylprednisolone 1 mg/Kg daily intravenously for 5 days, followed by 40 mg daily for 3 days and, lastly, 10 mg daily for 2 days, or dexamethasone 20 mg daily intravenously for 5 days, followed by 10 mg daily for 3 days and lastly 5 mg daily for 2 days) Consider ECMO in case of refractory hypoxemia despite invasive mechanical ventilation	Severe respiratory symptoms (ARDS)	**Remdesivir** with a loading dose of 200 mg intravenously followed by a maintenance dose of 100 mg/die intravenously for 10 days + **CQ phosphate** 500 mg BID for 20 days **or** **HCQ phosphate** 200 mg BID **or** **Lopinavir/ritonavir** 200/50 mg 2 tablets BID + **CQ phosphate** 500 mg BID for 20 days **or** **HCQ phosphate** 200 mg BID Alternative regimen: **darunavir/cobicistat** 800/150 mg QD (for 5 to 20 days, according to clinical evolution)In case of ARDS: **Dexamethasone** 20 mg/day for 5 days, then 10 mg/day for 5 days **and/or** **Tocilizumab** 8 mg/kg, maximum 3 infusions (maximum dosage per infusion 800 mg); second infusion after 8–12 h; in case of inadequate response eventually third infusion after 16–24 h	Required Intensive Unit Care

*Rheumatological drugs are reported in red*.

* *Alternatively to Lopinavir/ritonavir, Darunavir 600 mg tablets, 1 tablet q12 plus Ritonavir 100 mg tablets, 1 tablet q12, for 14 days*.

** *Before chloroquine and hydroxychloroquine administration, a G6PD deficiency test should be performed*.

° *Do not co-administrate Remdesivir with lopinavir/ritonavir, due to possible drug interactions*.

∧ *Tocilizumab administration should be guided by the presence of 1 or more of following selection criteria: a) PaO2/FiO2 ratio <300 mmHg; b) rapid worsening of respiratory gas exchange with or without the availability of non-invasive or invasive ventilation; c) IL-6 levels >40 pg/mL if not available, see D-dimer levels >1,000 ng/mL. Therapeutic schedule: 2 administrations (each 8 mg/kg, maximum 800 mg). Second administration at 8–12 h from the first one. Repeat C-reactive protein and D-dimer (±IL-6) after 24 h from each administration*.

*MWES, Modified Early Warning Score (0–2 stable, 3–4 unstable, ≥5 critical); HCQ, hydroxychloroquine; CQ, chloroquine; ARDS, acute respiratory distress syndrome; ECMO, extracorporeal membrane oxygenation; q, every; BID, bis in die (twice daily); QD, quam die (once daily); BCRSS, Brescia-COVID respiratory severity scale*.

### Chloroquine and Hydroxychloroquine

#### The Role in Rheumatic Diseases

When malaria was a major international problem for public health, causing millions of infections and deaths (74), CQ was the first adopted antimalarial drug. Due to the appearance of CQ-resistant Plasmodium falciparum strains, CQ has been gradually dismissed for malaria treatment, but it is currently used, together with, as an alternative, its hydroxy-analog HCQ in RA and several connective tissue diseases, although their mechanism of action is still largely unknown (75). Systematic reviews of randomized controlled and observational studies of antimalarial drugs in SLE strongly support the immunomodulatory capacity of HCQ, including the ability to prevent disease flares, promote long-term survival, and control disease activity during pregnancies without evidence of fetotoxic or embryotoxic effects (76, 77). Furthermore, in patients with SLE, HCQ can delay or prevent organ damage (78) and has shown antithrombotic effects (79). In the largest monocentric longitudinal study aimed at evaluating the safety profile of antimalarials involving 504 patients with SLE and discoid lupus erythematosus, the side effects were mild or moderate in most cases and were experienced by 19.3% of those treated with HCQ and 8.6% of those treated with CQ; maculopathy represented the main cause of treatment withdrawal (80). Despite the general HCQ/CQ acceptable safety profile, the possible risk of hemolytic effects in patients with glucose-6-phosphate dehydrogenase (G6PD) deficiency cannot be overlooked (81). In this regard, the occurrence of such complication in patients with COVID-19 treated with antimalarials has been just reported (82). In these cases, discontinuation of HCQ/CQ is advisable as the hemolysis is generally self-limiting once the anti-malarial has been withdrawn.

The anti-inflammatory properties of these drugs are supported by the results of *in vitro* studies demonstrating that CQ and HCQ equally reduce the secretion of some of the main pro-inflammatory cytokines, including TNF and IL-6, from peripheral blood mononuclear cells (83). Besides, CQ and HCQ accumulate in lysosomes and inhibit their function by increasing the pH, leading to an impairment of lymphocyte biological activity. Being involved in lysosome pH alteration, both CQ and HCQ can alter cells' autophagy with a consequent impact on their recycling and survival. During stressful conditions, autophagy can shape the adaptive immune response orchestrating the regulation of lymphocyte survival, differentiation, and activation (84). In autoimmune diseases, the deregulation of autophagy processes has been described (85). We demonstrated the role of this process in promoting both the exposure of immunogenic peptides (86) and the immune cell survival in patients with RA (87). Moreover, in patients with SLE, we showed a natural resistance of T lymphocytes to autophagy and up-regulation of genes, such as α-synuclein, able to negatively regulate this pathway (88, 89). The antiviral activity of antimalarials is further enhanced, at least *in vitro*, by the capacity to alter protein glycosylation including that of the viral envelop proteins, thus interfering with the virus assembly and release of mature virus particles (90).

#### The Role in Cardiovascular Risk and Coagulation

Apart from the anti-inflammatory and anti-viral properties, both CQ and HCQ interfere with hypercoagulation occurring in inflammatory states, directly impairing coagulators' function and thus preventing thrombotic events. In the context of rheumatic diseases, most evidence of their anti-thrombotic properties stems from APS, where HCQ is used both as primary (91) and/or secondary prophylaxis (92). Indeed, antiphospholipid antibodies (aPL) promote endothelial dysfunction, inflammation, and a marked pro-coagulant state. Although the precise mechanism of action is still unclear, HCQ administration in these patients seems to interfere with clots formations and with endothelial cells activation. To confirm, in cultured human endothelial glomerular cells, CQ prevented the expression of plasminogen activator inhibitor 1, an inhibitor of fibrinolysis (93). In APS, HCQ also reduces thrombin generation time and improves endothelial-dependent relaxation by modulating endothelial nitric oxide (NO) synthase and improving the production of NO (94). Accordingly, beneficial effects of HCQ on the endothelial dysfunction induced by oxidative stress were observed in APS mouse models (95). Evidence on interferences with tissue factor expression with consequent reduction of its soluble form further confirm HCQ ability to modulate endothelial cell activation (96). Finally, in APS, HCQ showed to reduce aPL titers with an apparent decrease in the incidence of arterial thrombosis (21). This finding is particularly remarkable if we consider that cases of COVID-19 patients testing positive for aPL antibodies have been described (21).

The anti-thrombotic properties of CQ have been demonstrated in conditions other than APS, including mice models of pancreatic adenocarcinoma, where CQ diminished the associated hypercoagulability by affecting neutrophil production of NETs, containing several procoagulant factors (97).

#### The Role in COVID-19 Patients

As above mentioned, antimalarials show the capacity to alter lysosome pH with consequent impairment of cells' autophagy properties. Lysosomes are involved not only in recycling cellular substrates but also in antigen processing and MHC class II presentation (98), which explains the antiviral activity of CQ, first demonstrated *in vitro* in 1969 (99). The increased local pH disrupts the function of several enzymes, including acid hydrolases, and inhibits the post-translational modification of newly synthesized proteins. By these properties, CQ and HCQ interfere with the endosome-mediated viral entry or with the later stages of replication of enveloped viruses (100). *In vitro* experiments performed on SARS-CoV demonstrated both protective effects of CQ in cells exposed to the virus and pre-treated with this drug and anti-viral effects in cells infected by CoV and subsequently treated with CQ (101). Likewise, HCQ inhibited *in vitro* SARS-CoV-2 infection, suggesting that this drug, also due to its anti-inflammatory function, has a good potential to combat the disease with less toxic effects compared to CQ (102, 103).

Results from more than 100 patients with COVID-19 demonstrated that CQ is superior to the control treatment in inhibiting the exacerbation of pneumonia, improving lung imaging findings, promoting a virus-negative conversion, and shortening the disease course in the absence of severe adverse reactions (104), although no data about clinical characteristics and demographics of both groups were reported.

Based on these preliminary observations, CQ and HCQ were introduced in the protocols for treating patients with COVID-19. According to pharmacokinetic models and to the most recent *in vitro* data, Xueting Y et al. recommend using HCQ in a loading dose of 400 mg twice daily for 1 day followed by a maintenance dose of 200 mg twice a day for 4 days. This dosing regimen allowed for an earlier (5 days in advance) and higher potency compared to CQ given 500 mg twice a day (105).

In an open-label non RCT involving 36 subjects with an upper or lower respiratory infection, HCQ treatment (600 mg daily) was significantly associated with viral load reduction/disappearance, especially when used by concomitant azithromycin (106), although the effect was purely microbiological and not clinical. A study from China seems to demonstrate a reduction in time to clinical response as well as a better progression of pneumonia in patients treated with HCQ in association with the standard of care compared to those not treated with HCQ (107), while another small Chinese pilot study showed no difference between HCQ-treated patients and a control group in terms of the negative conversion rate of pharyngeal swabs, duration of fever, and radiographic progression on CT chest images (108). Since then, more studies have been published dampening hopes on possible benefit of HCQ treatment in patients with COVID-19. Specifically, in patients requiring oxygen supplementation, HCQ at a dose of 600 mg/day within 48 h of admission to hospital did not produce a better outcome compared to standard care without HCQ. In particular, at day 21, no difference was identified in terms of overall survival rate, survival rate without transfer to the intensive care, and survival rate without ARDS (109). Another study on a larger group of patients (*n* = 1,376) does not support an association between HCQ administration and either a greatly lowered or an increased risk of the composite endpoint of intubation or death (110). Results from a new RCT aimed at clarifying whether combination therapy with azithromycin and HCQ can shorten hospitalization duration in COVID-19 patients are eagerly awaited (111). Although generally well-tolerated when used in autoimmune diseases, the different dosages of antimalarials in COVID-19 raise safety issues requiring a careful assessment.

Furthermore, registries of patients with COVID-19 and autoimmune rheumatic diseases have shown that ~25% of infected patients were already taking HCQ, indicating that this drug might not have a protective effect (112). In a small series of 17 SLE patients treated with HCQ (median/range 7.5/0.5–29.8 years), COVID-19 exerted as pneumonia in 13, respiratory failure in 11, and ARDS in five patients (113), making questionable the use of antimalarials as prophylactic treatment against this infection. While this remains a matter of debate (114), only rigorous and powered RCTs will uncover the uncertainty regarding the optimal use of antimalarials in COVID-19.

Currently, more than 200 clinical studies on the use of CQ and HCQ in COVID-19 are registered in ClinicalTrials.gov, and, on March 28th, 2020, the US Food and Drug Administration (FDA) gave an emergency use authorization for clinicians to prescribe CQ and HCQ in patients admitted to hospital, for COVID-19, even outside clinical trials, despite the presence of “limited *in-vitro* and anecdotal clinical data” (115).

In Italy, the National Institute for the Infectious Diseases “L. Spallanzani” recommends to treat COVID-19 patients presenting with respiratory and/or systemic symptoms with HCQ 400 mg, one tablet q12, as a loading dose, followed by 200 mg, 1 tablet q12, for 10 days or CQ 250 mg, two tablets q12, for 10 days after performing a G6PD deficiency test in combination with supportive and other anti-viral therapy. The same scheme should be applied to patients affected by respiratory symptoms who are clinically unstable and who are not in critical conditions as well as in critical patients. In these cases, HCQ/CQ should be combined with tocilizumab and supportive and other anti-viral therapy (72). Similar recommendations are provided by the Italian Society of Infectious and Tropical Diseases, Lombardy Region Section (https://www.simit.org/images/documenti/Linee%20guida%20SIMIT%20LAZIO%20SARS%20CoV%202%20maggio%202020.pdf) (Table 1).

Following the increasing use of antimalarials in COVID-19 patients, safety issues emerged about serious and, in some cases, fatal heart rhythm problems, particularly when CQ or HCQ were taken at high doses or in combination with azithromycin. Furthermore, empirical evidence from animal studies suggests that antimalarials may paradoxically increase the severity of some viral infections (chikungunya, dengue, and influenza), including those where inflammation sustains the disease pathology (116). On April 24, 2020, FDA issued a safety communication strongly encouraging close monitoring of patients in which antimalarials were used to prevent or treat COVID-19 to mitigate serious and potentially life-threatening heart rhythm problems. These data, together with the above controversial clinical reports, urge large, well-designed studies to make definitive conclusions.

## Other Rheumatologic Drugs of Potential Interest in the Treatment of COVID-19

### Janus Kinase Inhibitors

#### The Role in Rheumatic Diseases

JAKs are a family of non-receptor protein tyrosine kinases that affect intracellular signaling through their association with transcription factors known as STATs (signal transducers and activators of transcription), thereby forming the JAK/STAT pathway. JAKs are constitutively bound to their associated receptors on the cell surface and are activated when such receptors are engaged by their specific ligands, either cytokines, including IL-6 and IFN-α, β and γ family, or growth factors (117). In humans, the JAK family encompasses four members, comprising JAK1, JAK2, JAK3, and tyrosine kinase 2 (TYK2). JAK1, JAK2, and TYK2 are ubiquitously expressed in mammalian cells, whereas JAK3 is primarily expressed by cells of hematopoietic origin (118). Because JAK/STAT pathway is involved in signal transduction of different immunoregulatory cytokines, it also plays a pivotal role in the pathogenesis of different immune-mediated diseases, including RA (119), where JAK/STAT activation is associated with elevated levels of IL-6 (120). Recently, drugs inhibiting the JAK/STAT pathways named JAK inhibitors demonstrated efficacy in the treatment of different immune-mediated conditions (121) and, in the context of rheumatic diseases. In the last years, RA patients could benefit from the use of two different JAK inhibitors, both approved by EMA in 2017: tofacitinib, which is mainly a JAK 1/3 inhibitor, and baricitinib, which is mainly a JAK 1/2 inhibitor (30). Tofacitinib has been tested, in monotherapy or combination, in different clinical settings of RA: patients with an inadequate response to conventional treatment or biological agents and those naive for any treatment. Tofacitinib was effective in all of these conditions and exhibited a clinical response similar to or better than that of TNF antagonists, rapid onset of action, and generally a sustained effect (122, 123). Similarly, baricitinib in different clinical settings showed rapid and sustained therapeutic efficacy in RA patients (124, 125). In 2019, EMA approved a new JAK-1 selective inhibitor, named upadacitinib, for the use in RA following the encouraging data in patient's refractory to other biological therapies (126) or as monotherapy in non-responders to methotrexate (127).

Despite differences in selectivity between JAK inhibitors, a large overlap exists in their safety profiles with regards to increased risk of infections, drop in blood cell count, and increase in vascular events. Interestingly, apart from bacterial infections, patients treated with JAK inhibitors are typically at risk for viral infections, including the reactivation of the varicella-zoster virus and, to a lesser extent, cytomegalovirus infections (128). This may be advocated to JAK inhibitors targeting NK cell activation and anti-viral immunity, especially IFN-α, β, and γ, which have well-known potent antiviral effects.

Finally, as above mentioned, ILD may be found in RA patients, but data on the efficacy of JAK inhibitors in these specific manifestations are still lacking (129). Nonetheless, tofacitinib successfully controlled acute pulmonary involvement in a patient with dermatomyositis (130) and suppressed the progression of the disease in mice models of ILD (131), opening up for new perspectives on the possible efficacy of this therapy also in inflammatory lung conditions.

#### The Role in Cardiovascular Risk and Coagulation

Hyperactive JAK-signaling also critically influences coagulation and thrombosis. The huge anti-inflammatory activity displayed by JAK inhibitors probably represents the main protective ability toward the hyper-coagulation occurring in COVID-19. Indeed, the inhibition of JAK-mediated signaling involving different pro-inflammatory cytokines, including IL-6, would favor the recovery of the balance between anti- and procoagulant factors. Nonetheless, other issues need to be considered.

JAK2 is essential for the normal development of erythrocytes, granulocytes, and platelets, and its mutations can act as central drivers of myeloproliferative neoplasia. The use of JAK2 inhibitors to prevent thrombotic complications in myeloproliferative diseases is currently accepted (132). Specific JAK2 mutations (i.e., JAK2 V617F) can increase procoagulant activity in certain hematologic conditions characterized by increased thrombotic risk, such as polycythemia vera (PV), essential thrombocythaemia, and primary myelofibrosis (133). In PV or myelofibrosis, ruxolitinib (JAK1/2 inhibitor) decreased the risk of arterial and/or venous thrombosis (134). Thus, it might be reasonable to hypothesize a benefit of JAK inhibitors in hyper-inflammatory states accompanied by thrombocytosis and, accordingly, increased risk of hyper-coagulation.

In rheumatic conditions, despite the evidence of an acceptable safety profile (129), the possibility to develop iatrogenic cardiovascular complications is questioned. In 2017, an increased risk of deep vein thrombosis and pulmonary embolism in patients with RA treated with baricitinib has been reported (135). Nonetheless, data from subsequent studies allowed reconsidering these events estimating a thromboembolic risk of approximately five events per 1,000 patient years (136). Following data on baricitinib, the Federal Drug Administration Adverse Event Reporting System also raised concerns about a possible increased risk of pulmonary thrombosis in patients treated with tofacitinib (136), mainly associated with a high dosage (10 mg twice daily), which is currently not used in patients with RA (137). Subsequent studies showed a numerically higher, but statistically non-significant, risk of venous thromboembolism in RA patients treated with tofacitinib compared to those treated with TNF inhibitors (138).

Furthermore, there is evidence to suggest that JAK1/3 inhibition is responsible for raises in LDL and HDL cholesterol levels in patients with RA (139).

Despite concerns related to the possible thromboembolic risk, the use of JAK inhibitors in patients with COVID-19 could be overall beneficial. Indeed, the ischemic complications occurring in severe COVID-19 are mainly related to a local formation of thrombi rather than emboli and this is due to endothelial cell activation and an inflammatory-related procoagulant state where JAK inhibitors would likely display beneficial effects.

#### The Role in COVID-19 Patients

The potential utility of JAK inhibitors in COVID-19 patients has been suggested (140). Baricitinib has been proposed as part of the treatment of COVID-19 pneumonia due to both anti-inflammatory properties and the capability of impairing endocytosis, which is necessary for viral entry in the cells (141). Indeed, baricitinib binds the cyclin G-associated kinase, a regulator of endocytosis (142), but, most importantly, it is also a potent inhibitor of the numb-associated kinase (NAK) family with a particularly high affinity for AP2-associated protein kinase 1 (AAK1), a pivotal regulator of clathrin-mediated endocytosis (CME). This is the major endocytic pathway responsible for the uptake of transmembrane receptors and transporters, for remodeling plasma membrane composition in response to environmental changes, and for regulating cell surface signaling (143). Not surprisingly, CME is also implicated in cell virus infections. Compared to the other JAK 2 inhibitors, such as fedratinib (a selective JAK 2 inhibitor) and ruxolitinib (JAK 1/2 inhibitor), baricitinib is the most likely inhibitor of CME. Specifically, the predicted unbound plasma exposure required to inhibit the enzymes needed for CME greatly exceeds the currently tolerated dosages proposed for fedratinib and ruxolitinib. By contrast, at therapeutic dosing for RA treatment (either as 2 mg or 4 mg once daily), the free plasma concentrations of baricitinib are predicted to be sufficient to inhibit AAK1 (141).

The use of baricitinib in patients with mild to moderate COVID-19 is currently under evaluation in 13 different clinical studies (ClinicalTrials.gov). The efficacy of ruxolitinib will be evaluated as well in patients with SARS-related to COVID-19 in 14 different clinical studies worldwide. Among them, a study promoted by Novartis (ClinicalTrials.gov: NCT04337359) will be performed with the purpose to allow access to ruxolitinib for eligible patients with severe to very severe COVID-19. Two clinical trials evaluating the use of tofacitinib both as an early treatment in patients with symptomatic pneumonia and in combination with HCQ vs. HCQ alone are currently ongoing in Italy (ClinicalTrials.gov: NCT04332042, NCT04390061). Despite the rationale for its use in COVID-19, no study is currently evaluating upadacitinib in this condition.

### IL-1 Inhibitors

#### The Role in Rheumatic Diseases

IL-1 is part of a family embracing 11 members, the most studied of which are the pro-inflammatory pyrogen cytokines IL-1α and IL-1β and the anti-inflammatory IL-1 receptor antagonist (IL-1Ra). IL-1α and IL-1β bind the type 1 IL-1 receptor (IL-1R1) on responsive cells, triggering a cascade of signaling events that boost the inflammatory response (144). IL-1Ra is a naturally occurring glycoprotein inhibitor of IL-1 that binds the high-affinity cell surface IL-1R but has no receptor activation function (145). The agonist effects of IL-1 are therefore partially regulated by IL-1Ra. The pro-inflammatory activity of IL-1 is particularly overt following the trigger of the NLRP3 inflammasome, a major intracellular multiprotein implicated in caspase-1 activation and ultimately in the production of two major innate immune mediators: IL-1β and IL-18 (146). The dysfunction of NLRP3 inflammasome activation is implicated in many of the so-called autoinflammatory syndromes (147).

In March 2002, anakinra, a recombinant form of IL-1Ra, was one of the first biological agents approved for the treatment of RA in Europe. Since then, anakinra obtained further marketing authorization for some autoinflammatory syndromes, such as cryopirinopathies (CAPS) and AOSD, both of which may be complicated by ARDS or SIRS, while canakinumab, a novel human monoclonal antibody targeting IL-1β, is now approved for CAPS, AOSD, and gout.

The availability of more efficacious biological agents for RA has over time greatly diluted the use of anakinra, but the acknowledgment of the crucial role of IL-1 in other conditions, including type 2 diabetes (T2D), atherosclerosis, and acute myocardial infarction (148), aroused a renewed interest in the use of this drug for patients with RA and comorbidities (149).

Apart from RA, anakinra and, more recently, canakinumab, have been widely used in AOSD patients with brilliant results on the most typical manifestations of the disease, including fever, rash, sore throat, hyperferritinemia, lymphadenopathies, and increased liver enzymes. Specifically, in 140 patients with active AOSD, anakinra proved to be effective in reducing all clinical and serological manifestations within a few days from the first administration, and primary and secondary inefficacy was only reported in 10.7 and 7.8% of patients, respectively (150). Of interest, the development of MAS is a major life-threatening complication in AOSD (151). Similar to ARDS and SIRS, MAS is mediated by a cytokine storm possibly followed by SIRS and multiple organ failure. In these cases, IL-1 inhibitors provided excellent results, further supporting their use in AOSD treatment (152). Both anakinra and canakinumab display a good safety profile. The most frequent adverse event accompanying the treatment with anakinra is represented by injection site reactions, while both anakinra and canakinumab may favor infections (manly mild upper airway infections), elevated liver enzymes, mild leukopenia, and myopathy (152).

#### The Role in Pulmonary Inflammation

Even if ARDS can be a life-threatening complication for patients with AOSD (153–156), there is no available evidence supporting the efficacy of IL-1 inhibition in such situations. However, it is reasonable that IL-1β and IL-18 may have a prominent role in acute lung injury possibly linked to inflammasome activation mediated by both infectious stimuli and mechanic ventilation (157). In a mouse model of acute lung injury, IL-1β was detectable in BAL fluids in a macrophage- and neutrophil-dependent manner; additionally, neutrophil-derived extracellular histones directly activated the NLRP3 inflammasome (158). Interestingly, inflammasome activation is also involved in chronic lung diseases such as asthma and COPD (159). These data are in agreement with subsequent *in vitro* studies demonstrating an epithelial repair effect by IL-1β (160).

Anakinra has been used in bleomycin-induced models of acute lung injury, demonstrating the capability to reduce lung neutrophil infiltration and cytokine levels in BAL fluid (161). Accordingly, neutralization of IL-1β as well as administration of IL-1Ra seems able to attenuate acute lung injury in mice (157, 162). On the other hand, early studies demonstrated that IL-1Ra is elevated in plasma and BAL fluid of ARDS patients and is associated with disease outcome (163). Likewise, in pediatric ARDS, an association between IL-1Ra serum levels and the length of mechanical ventilation and mortality has been demonstrated (164). Finally, in patients with community-acquired pneumonia, a specific polymorphism of IL1-Ra seems associated with adverse outcomes and higher IL1-Ra serum levels (165).

#### The Role in Cardiovascular Risk and Coagulation

A bi-directional relationship also exists between IL-1-mediated inflammation and coagulation. Indeed, similarly to IL-6, IL-1 concurs in the alteration of the balance between pro-thrombotic and anti-thrombotic mechanisms. As mentioned above, Il-1 seems to maintain thrombosis by increasing the time of clot lysis (59). There seems also to be a strict relationship between platelet reaction and IL-1β production. Specifically, both the platelet number and their degranulation activity are associated with IL-1β plasma concentration (166). Accordingly, in patients with SLE, endothelium activation seems mediated by activated platelets via an IL-1β pathway (167) and, in a mouse model of DIC, IL-1β could upregulate the expression of tissue factor, favoring the generation of intravascular thrombi (168). In patients with AOSD DIC, frequently associated with MAS, has been successfully treated with anakinra (169, 170). In line with this evidence, an IL-1 receptor blockade was found associated with significant improvement in patients' survival also in DIC-associated sepsis (171).

To date, most data regarding the beneficial effects of IL-1 inhibition in cardiovascular events come from experiences on atherosclerosis and ischemic heart disease. Increasing evidence supports a major role for therapies targeting IL-1 in the prevention of cardiovascular events (172). IL-1 promotes the formation, growth, and rupture of vascular atherosclerotic plaques, and both IL-1β and IL-1α are highly expressed in atherosclerotic lesions, promoting the recruitment of leukocytes by inducing the expression of adhesion molecules in endothelial cells (173). Complex plaques seem to produce great amounts of IL-1β, supporting the idea that inflammasome is the main pathway for IL-1α/β generation in atherosclerosis (174). Compared to normal arteries, expression of NLRP3, as well as ASC proteins, caspase-1, IL-1β, and IL-18 mRNA is significantly increased in atherosclerotic plaques, and specific genetic variants seem associated with the pathogenesis of atherosclerosis (175).

Inhibition of IL-1 mediated inflammation by anakinra is also effective in acute myocardial infarction with consequent evidence of a reduction in the development of heart failure (176). The massive Canakinumab Anti-inflammatory Thrombosis Outcomes Study (CANTOS) confirmed the protective effects of IL-1β inhibition in patients with prior myocardial infarction and evidence of systemic inflammation underlining the reduction of recurrent nonfatal myocardial infarction, non-fatal stroke, cardiovascular death, and reduced need for coronary revascularization (177). Emerging evidence also demonstrates brilliant results of anakinra treatment in myocarditis and dilated cardiomyopathies (178, 179).

#### The Role in COVID-19 Patients

Even if the evidence shows an ambivalent role of IL-1 in lung inflammation and data on the efficacy of IL-1 inhibition in humans with ARDS are scant, the blockage of IL-1 in severely ill patients with COVID-19 remains appealing. This approach would be particularly advisable for COVID-19 patients experiencing a MAS-like syndrome related to the cytokine storm. In these cases, continuous infusion of anakinra may result in rapid serologic and subsequent clinical improvement (180). Indeed, a recent retrospective cohort study demonstrated a significant amelioration of inflammatory parameters and respiratory function in 29 Italian patients treated with high dosage of intravenous anakinra (5 mg/kg twice a day) in association with non-invasive ventilation and standard therapy (anti-viral drugs and HCQ). Specifically, a significant improvement of survival rate was demonstrated in patients treated with anakinra compared to controls (181). To date, this is the only large study on the use of anakinra evaluating patients with severe ARDS and hyper-inflammation associated with COVID-19. In parallel, a small open label study performed in France confirmed the efficacy of subcutaneous administration of anakinra (100 mg twice a day) in eight out of nine patients with moderate to severe COVID-19 at high risk of worsening (182). Data on the early us of anakinra in COVID-19 patients are also available. Specifically, a rapid resolution of systemic inflammation and remarkable improvement of respiratory parameters was demonstrated in five patients with early signs of COVID-19 treated with high dose of intravenous anakinra added to the current standard of care (100 mg every 8 h for 24–48 h, followed by tapering according to clinical response) (183). Finally, the efficacy of high intravenous dosage of anakinra has been tested in patients treated in the intensive care unit and complicated with secondary hemophagocytic lymphohistiocytosis (sHLH). Although three patients died, the mortality rate in this small cohort study was lower than historical series of patients with sHLH in sepsis dysfunction; decreased needs for vasopressors, improved respiratory function, and lower Hemophagocytosis Score were also demonstrated (184).

A phase 2/3 RCT on the use of anakinra in patients with SARS-CoV-2 infection has just started in Italy to investigate new possibilities to reduce the requirement for mechanical ventilation. Specifically, three arms of treatment will be set, including anakinra in combination with the standard of care, emapalumab (a monoclonal antibody blocking IFN-γ) in combination with the standard of care, and the standard of care alone (ClinicalTrials.gov: NCT04324021). The efficacy of anakinra in COVID-19 severe patients is also under evaluation in 13 other different clinical studies from Europe, USA and Australia (ClinicalTrials.gov). Additionally, three different clinical studies evaluating the efficacy of canakinumab in patients with COVID-19 have been planned; two of them will soon be started in Italy and the USA, the other one is promoted by Novartis (NCT04362813).

## Conclusions

At the time we are writing this review, with the SARS-CoV-2 infection increasingly spreading worldwide, about 300 trials out of 1,684 studies registered in ClinicalTrials.gov involve drugs used in immune-rheumatologic diseases, some of which directed against cytokines pivotal for the pathogenic processes both in autoimmune/inflammatory rheumatic diseases and in SARS. Old and new agents offer now hope in the treatment of the complications of COVID-19. In this review, we have presented evidence for the rationale of their application in this threatening infectious condition.

## Author Contributions

SC, RS, and RP defined the content of the manuscript. SC, RS, and CB contributed to literature search and manuscript writing. FC contributed defining the contentment of the manuscript and revised it together with RP. All authors approved the final version of the manuscript.

## Conflict of Interest

The authors declare that the research was conducted in the absence of any commercial or financial relationships that could be construed as a potential conflict of interest.
